# Chicago Medical Society

**Published:** 1869-07-01

**Authors:** Hiram Wanzer

**Affiliations:** Secretary


### SOCIETY REPORTS.

### *Chicago Medical Society.*

FRIDAY EVENING, May 28, 1869.

Regular weekly meeting of Chicago Medical Society, President Bogue in the chair. Proposals for new members being in order, Dr. A. I. Brackett was proposed by Drs. Schmidt and Paoli, and referred to board of censors. Discussion for the evening being now in order, viz., "What is the best treatment of Leucorrhœa," Dr. Trediche stated the disease might arise from a cancerous or syphilitic diathesis; the discharge might proceed from the cavity of the fundus body or neck of the uterus—from the vagina or pudenda; the disease might be functional or inflammatory; acute or chronic. Treatment: where there is plethora, saline purgatives, or abstraction of blood. Mild vaginal enæmas are best adapted in the acute form. In the chronic variety, where the discharge is excessive from the uterus, both constitutional and local measures are indicated, viz., ferruginous tonics and supporting diet, opium, conium, and hyoscyamus to allay pain. Locally, argent nitras in solution or solid stick to the part affected; decoctions oak bark and blisters to the sacrum, where the cause was displacement or procedentia uteri, to reposit the organ. Dr. Merriman stated leucorrhœa was catarrhal in its nature, and requires constitutional treatment; that urethal, etc., discharge proceeds from the cavity of the uterus or vagina; that it should be treated as a nervous disease: iron, which is an astringent, also strychnine, nux vomica and arsenic, which have control over the peripheral, nervous filaments and arterioles; that the treatment should be persistent. As the disease becomes local the most soothing medicines are indicated, viz., the warm sitz baths, vaginal anodynes and astringent enæmas, and insufflation of the powder of bismuth. Dr. Gray believes it a local disease, and has treated it as such with success. Dr. Foster stated that in mechanical displacement of the organ, giving rise to leucorrhœa, to reposit the organ was the only remedy, that where the disease approaches gonorrhœa local anodynes and astringents cure. Dr. Mitchell stated where the disease was incident to those local affections of the skin, arsenic was a good remedy. Dr. Paoli stated leucorrhœa was not a disease but a symptom; that we seldom saw it in the acute form; that the chronic variety was most persistent. The patient is generally anemic, and prone to pulmonary affections; that the symptoms follow frequent abortions, metritis and mechanical displacements, procidentia uteri, etc. Dr. Fitch stated the disease may be either local or constitutional and should be treated accordingly; that there was a want of tonicity in the system, and a watery condition of the blood. The constitutional treatment should consist of iron, general tonics and liberal diet. In cervical discharges prefers the local use of solid argentinitras within the cervix, but never injections into the uterine cavity; where discharges were offensive had used vaginal enæmas of solution permanganate of potassa. Society adjourned.

HIRAM WANZER, *Secretary.*

---

